# John Ensor La Motte

**Published:** 1878-03

**Authors:** 


*John Ensor La Motte, D. D. S.*—Those who were associated with the above-named gentleman in the classes of 1876-7 of the Baltimore College of Dental Surgery, will deeply regret to know that he died of phthisis pulmonalis, at his residence, Hampstead, Carroll County, Md., February 24th, aged 27 years.

Of genial, winning manners, and strictly moral habits, Dr. La Motte greatly endeared himself to the teachers under whose instruction he had placed himself; and he no less popular with the class, at whose head he stood by common consent as an operator. He graduated with peculiar honor, and stood fair to achieve eminence in his chosen profession. Gentle in his manners, pure in his morals, he made friends wherever he chanced to be. He bore his sickness with great fortitude, and with resignation saw its fatal termination approach; and though expressing himself as having hoped to win an honorable name in life, he turned without regret to the hope of a future better existence. H.

